# Essentials of Nervous Diseases and Insanity

**Published:** 1893-05

**Authors:** 


ESSENTIAL OF NERVOUS DISEASES AND INSANITY — THEIR SYMPTOMS AND TREATMENT—A MANUAL FOR STUDENTS AND PRACTITIONERS, by John C. Shaw, M. D., Clinical Professor of Diseases of Urine and Nervous System Long Island Hospital Medical College, etc., etc., etc. Published by W. B. Saunders, 913 Walnut St., Philadelphia.

This compendium, unlike most others, is worthy the time necessary to read it. The method and expression of the author are admirable. Much work has been spent thereon. The cuts are good and the descriptions as full as the aims of the author claim. H. H.

